# Intranasal Administration of Recombinant *Mycobacterium smegmatis* Inducing IL-17A Autoantibody Attenuates Airway Inflammation in a Murine Model of Allergic Asthma

**DOI:** 10.1371/journal.pone.0151581

**Published:** 2016-03-14

**Authors:** Wanting Xu, Ling Chen, Sheng Guo, Liangxia Wu, Jianhua Zhang

**Affiliations:** 1 Department of Paediatrics, Shanghai Jiao Tong University Affiliated Sixth People’s Hospital, Shanghai, China; 2 Department of Endocrinology, Shanghai Jiao Tong University Affiliated Children’s Hospital, Shanghai, China; Baylor Institute for Immunology Research, UNITED STATES

## Abstract

Asthma is a chronic inflammatory disorder, previous studies have shown that IL-17A contributes to the development of asthma, and there is a positive correlation between the level of IL-17A and the severity of disease. Here, we constructed recombinant *Mycobacterium smegmatis* expressing fusion protein Ag85A-IL-17A (rMS-Ag85a-IL-17a) and evaluated whether it could attenuate allergic airway inflammation, and further investigated the underlying mechanism. In this work, the murine model of asthma was established with ovalbumin, and mice were intranasally vaccinated with rMS-Ag85a-IL-17a. Autoantibody of IL-17A in sera was detected, and the airway inflammatory cells infiltration, the local cytokines and chemokines production and the histopathological changes of lung tissue were investigated. We found that the administration of rMS-Ag85a-IL-17a induced the autoantibody of IL-17A in sera. The vaccination of rMS-Ag85a-IL-17a remarkably reduced the infiltration of inflammatory cells and the secretion of mucus in lung tissue and significantly decreased the numbers of the total cells, eosinophils and neutrophils in BALF. Th1 cells count in spleen, Th1 cytokine levels in BALF and supernatant of splenocytes and mediastinal lymph nodes, and T-bet mRNA in lung tissue were significantly increased with rMS-Ag85a-IL-17a administration. Meanwhile, rMS-Ag85a-IL-17a vaccination markedly decreased Th2 cells count, Th2 cytokine and Th17 cytokine levels in BALF and supernatant of splenocytes and mediastinal lymph nodes, and chemokines mRNA expression in lung tissue. These data confirmed that recombinant *Mycobacterium smegmatis in vivo* could induce autoantibody of IL-17A, which attenuated asthmatic airway inflammation.

## Introduction

Asthma is a chronic inflammatory disease characterized by episodic airway hyperresponsiveness, mucus gland hyperplasia and reversible airway obstruction, affecting millions of individuals all over the world [1–3]. It is well established that asthmatic airway inflammation associated with Th1/Th2 immune dysregulation [4, 5], The cytokines IL-4, IL-5 and IL-13 produced by Th2 cells are known to play important roles in asthma pathogenesis, particularly in eosinophilic asthma, they are critical for the accumulation of eosinophil and induction of immunoglobulin class switching to IgE [6, 7], meanwhile, they participate in mucus hypersecretion and airway hyperresponsiveness in asthma [8, 9].

In addition to the Th2 cytokines, attention has focused on Th17 cytokines as candidate drivers of severe asthma. Increasing evidence indicates that IL-17A is an important contributor to the development of asthma, especially to severe asthma characterized by airway intense neutrophil infiltration and less responsive to corticosteroids [10–12]. Clinical data show that IL-17A in sputum, bronchoalveolar lavage fluid (BALF) and sera of asthmatic patients is significantly increased compared to control subjects, and moreover there is a positive correlation between the level of IL-17A and the severity of disease [13, 14], underscoring the necessity to discover new strategies designed to suppress IL-17A. Anti-IL-17A monoclonal antibodies have been used to passively immunize asthmatic mice, and the results showed that although the monoclonal antibodies reduced airway neutrophil infiltration, Th2 cytokines such as IL-5 and IL-13 in BALF of mice were significantly increased [15, 16]. Hence, we speculate that autoantibody of IL-17A induced *in vivo* might avoid these weakness and be beneficial to asthma treatment.

Under normal conditions, self-antigen could not induce autoantibody due to immunological tolerance. According to the theory of Delavallée [17], in order to induce a B cell response and obtain autoantibodies to neutralize self-cytokines, cytokines should be modified with foreign Th cell epitopes. The Bacille Calmette-Guérin (BCG) vaccine has demonstrated marked immunomodulatory effects, as an immunodominant antigen of BCG and a major portion of *Mycobacterium* tuberculosis filtrate antigen, Ag85A is known to enhance Th1 cytokines response and includes several Th cell epitopes [18, 19]. *Mycobacterium smegmatis* (MS) is a non-pathogenic species of the mycobacteria family that presents a number of properties that make it an effective vaccine vector [20–22]. At present, recombinant *Mycobacterium smegmatis* vaccine has been widely used against tuberculosis, helicobacter pylori (HP) infection, hepatitis B virus (HBV) infection, parasitic infections and some cancers [23, 24].

In the present study, based on the hypothesis that Ag85A may serve as a foreign Th cell epitopes for inducing IL-17A autoantibody, we constructed a recombinant *Mycobacterium smegmatis* rMS-Ag85a-IL-17a (rMS). The efficacy of rMS in reducing asthmatic airway inflammation was evaluated and the potential related mechanism was further investigated.

## Materials and Methods

### Animals

Female BALB/c mice at 6–8 weeks of age and weighing 16–18 g were purchased from B&K Universal Group Ltd. (Shanghai, China). Mice were bred and maintained in a specified pathogen-free (SPF) laboratory animal facility, and the serology reports during the period of the whole experiment were negative for the presence of *Mycobacterium smegmatis*. The physiological condition of mice was checked three times a week before OVA intraperitoneal injection, and every day after OVA sensitization. An euthanasia protocol using ketamine was executed to minimize suffering and distress of mice, and no seriously ill or dead mice were found before sacrifice. The use of experimental animals was in accordance with the Institutional Animal Care and Use Committee (IACUC) of Shanghai Public Health Clinical Center, the IACUC approved the present study (Permit Number: 2013–102).

### Preparation of bacteria

The rMS-Ag85a-IL-17a (hereinafter referred to as rMS) expressing fusion protein Ag85A-IL-17A was constructed from *Mycobacterium smegmatis* in our preliminary experiments [25]. Briefly, a recombinant plasmid pMFA42S-Ag85a-IL-17a was constructed by inserting fusion gene Ag85a-IL-17a into shuttle vector pMFA42S, which was transformed to *Mycobacterium smegmatis* by electroporation to obtain recombinant *Mycobacterium smegmatis*. rMS transformants were grown on Middlebrook 7H11 solid agar containing 0.5% (v/v) glycerol, 10% (v/v) oleic acid albumin dextrose catalase (OADC, Difco) and 20 μg/ml kanamycin, and cultures were frozen in the exponential phase and stored at -80°C in 7H9 liquid broth containing 15% (v/v) glycerol, 10% (v/v) OADC, 0.05% (w/v) Tween-80 (Sigma-Aldrich) and 20 μg/ml kanamycin. Before vaccination, the rMS was recovered from stock cultures and diluted to the appropriate concentration with sterile phosphate-buffered saline.

### Immunization, sensitization and challenge protocols

**Fig 1:**
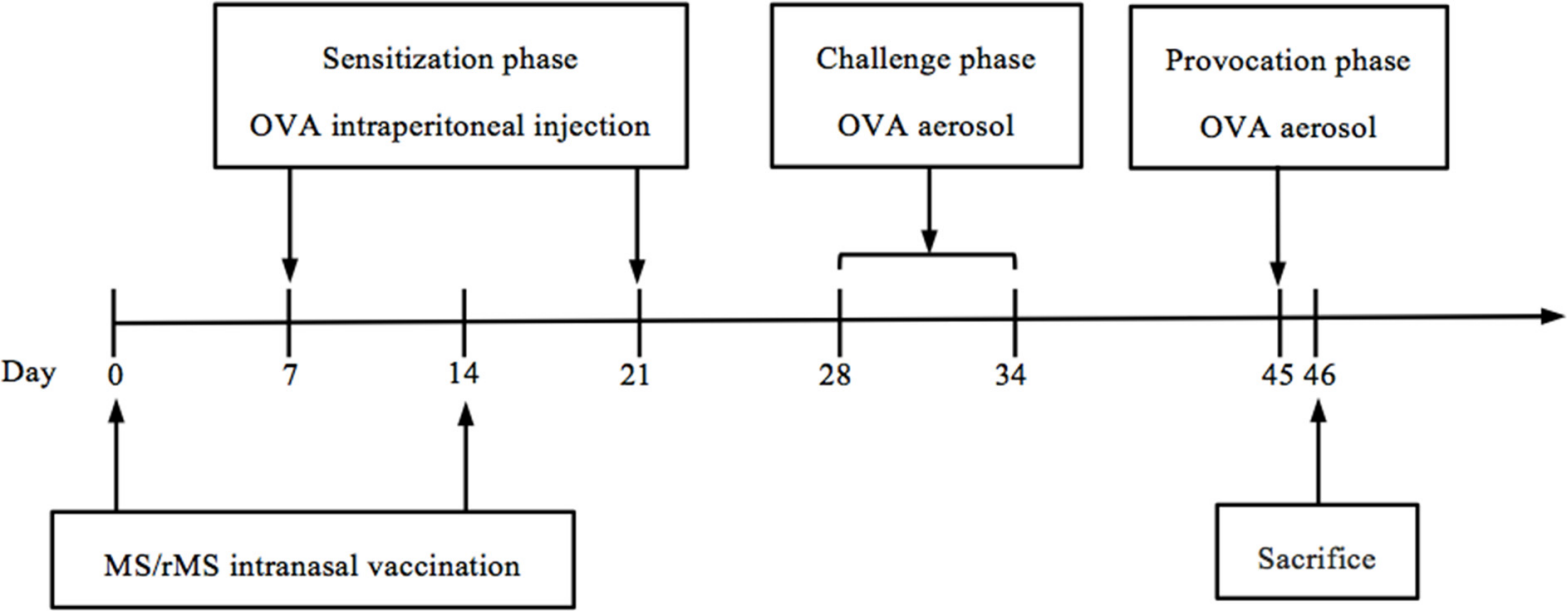
Animal immunization schedule. Mice were randomly divided into 5 groups and treated as shown above. Mice under light anesthesia in MS and rMS groups were administered intranasally with bacterial solutions on day 0 and day 14. Except for PBS group, all of mice were sensitized twice by intraperitoneal injections with OVA on day 7 and day 21. Seven days after sensitization, mice were challenged with nebulized OVA for seven consecutive days. Ten days after aerosol challenge, mice in severe asthma, MS and rMS groups were challenged again with OVA aerosol. Mice in PBS group were immunized, sensitized and challenged with PBS as parallel manipulation.

Mice were randomly divided into 5 groups of 6 animals (PBS, asthma, severe asthma, severe asthma plus MS and severe asthma plus rMS) and treated as shown in (Fig 1). Briefly, mice in MS group and rMS group under light anesthesia were administered intranasally with MS or rMS at a dose 1.0×10^7^ colony-forming unit (CFU) in 20 μl of phosphate buffered saline (PBS) on day 0 and day 14. Except for PBS group, all of mice were sensitized twice by intraperitoneal (*i*.*p*.) injections with 10 μg of ovalbumin (OVA, Grade V; Sigma-Aldrich) absorbed on aluminum hydroxide gel in 200 μl of PBS on days 7 and 21. Seven days after last sensitization, mice were challenged (20 min) with OVA aerosol generated by ultrasonic nebulization of a 1.0% (w/v) OVA solution in PBS for seven consecutive days to induce allergic airway inflammation. Ten days after aerosol challenge, mice in severe asthma with or without vaccination were aerosol challenged again with ultrasonic nebulization of a 20.0% (w/v) OVA solution in PBS for 20 min. Mice in PBS group were immunized, sensitized and challenged with PBS as parallel manipulation.

### Anti-IL-17A autoantibody assay in sera

Mice were sacrificed 24 h after the last OVA challenge. Blood was allowed to clot at room temperature, and centrifuged at 4,000 g for 10 min. Sera level of IL-17A specific IgG were measured by enzyme-linked immunosorbent assay (ELISA). In brief, Plates were coated with commercial recombinant protein IL-17A (2.5 μg/ml, PeproTech) and antibodies in sera were detected using goat anti-mouse IgG-HRP (1:5,000, Santa Cruz). Substrate solution (eBioscience) was added to each well and incubated for 10 min and reactions were stopped with 2 M H_2_SO_4_. Data are presented as mean of optical density (OD) value at 450 nm per group.

### Bronchoalveolar lavage (BAL) collection

Bronchoalveolar lavage was performed as described previously [26]. Briefly, the trachea was cannulated, BALF were prepared by washing the lungs three times with 0.4 ml ice-cold PBS. The cells were sedimented by centrifugation at 1,000 g for 10 min at 4°C. The supernatant (cell-free BALF) of the first lavage was stored at -80°C for cytokine analysis. The number of total leukocytes in BALF was determined using a hemocytometer. Differential cell counts were conducted by using Wright-Giemsa stained cytospin preparations in a blinded manner. At least 100 cells were counted twice and differentiated according to standard morphologic criteria under light microscopy.

### Preparation and culture of splenocytes

Spleens were isolated aseptically and cells were prepared by passage through 200 μm nylon net. Cells were washed with PBS by centrifugation at 800 g for 10 min, and erythrocytes were depleted using lysing buffer (Sigma-Aldrich). Following washing and centrifugation, cells were resuspended at 1.0×10^7^/ml for assay. One million cells were cultured in RPMI-1640 medium (GIBCO) supplemented with 10% (v/v) fetal bovine serum (FBS; GIBCO) and antibiotics (100 U/ml penicillin and 100 μg/ml streptomycin) and stimulated with OVA (10 μg/ml; grade V, Sigma-Aldrich) for 24 h at 37°C in the humidified incubator with 5% (v/v) CO_2_. Culture supernatants were collected after 24 h culture and centrifuged for 5 min at 1,000 g and the suspension was collected for cytokine analysis.

### Flow cytometric analysis

Flow cytometry was performed after stimulation of 10^7^ splenocytes with OVA (10 μg/ml) for 24 h at 37°C in 5% CO_2_. Cells were further incubated with GolgiPlug (0.5 μg/10^6^ cells, BD Pharmingen) to block cytokine secretion for 6 h at 37°C in 5% CO2. Cells were fixed and permeabilized with Cytofix/Cytoperm kit (BD Biosciences) at 4°C for 15 min according to the manufacture’s instructions. Cell staining was performed with antibodies conjugated with extracellular epitopes (CD3-PerCP-Cy5.5 and CD4-FITC, eBioscience) or intracellular cytokines (IFN-γ-APC and IL-4-PE, eBioscience) for 30 min at 4°C, and washed with PBS. Samples were run on a FACSCalibur (BD Biosciences) cytometer, cell selection after flow cytometry acquisition of 10,000 events was based on size (FSC) and granularity (SSC) to gate lymphocytes and analyzed with FlowJo software (TreeStar).

### Cytokine levels in BALF and culture supernatant of splenocytes

ELISA was performed according to the manufacturer’s instructions. The concentrations of cytokine in BALF and culture supernatant of splenocytes and mediastinal lymph nodes were measured using specific mouse IL-5, IL-6, IL-10, IL-12, IL-13 and IL-17A ELISA kits (eBioscience).

### Quantitative RT-PCR analysis

The mRNA expression levels were assessed by quantitative real-time polymerase chain reaction (RT-PCR). Briefly, lung tissues were removed from individual mice and homogenized mechanically by variable speed homogenizer, and total RNA was isolated from homogenized lungs using Trizol reagent (Invitrogen). One microgram of total RNA was reverse transcribed with Oligo (dT)_20_ primer. RT-PCR was performed using SYBR Green Real time PCR Master Mix-Plus kit (Takara). Primer sequences used in PCR amplification are showed in (Table 1). The data were normalized to GAPDH gene expression as a reference. Fold changes were calculated using the 2^-△△Ct^ statistical method [27].

**Table 1:** Primer sequences for quantitative real-time PCR.

Genes	Accession No.	Sequences (5'→3')	Length (bp)
GAPDH	NM_008084.2	Forward: TGCAGTGGCAAAGTGGAGATTGTTG	175
		Reverse: GGTCTCGCTCCTGGAAGATGGTGAT	
IL-6	NM_031168.1	Forward: GGCCTTCCCTACTTCACAAG	126
		Reverse: ATTTCCACGATTTCCCAGAG	
IL-10	NM_010548.1	Forward: GGTTGCCAAGCCTTATCGGA	191
		Reverse: ACCTGCTCCACTGCCTTGCT	
IL-23	NM_031252.2	Forward: CATGGGGCTATCAGGGAGTA	168
		Reverse: AATAATGTGCCCCGTATCCA	
Eotaxin-1	NM_011330.3	Forward: CTTCTATTCCTGCTGCTCACG	131
		Reverse: TTGTAGCTCTTCAGTAGTGTGTTGG	
MIP-2	NM_009140.2	Forward: CACCAACCACCAGGCTACAGGG	191
		Reverse: GGGCTTCAGGGTCAAGGCAAAC	
TGF-β	NM_011577.1	Forward: CGCAACAACGCCATCTA	303
		Reverse: GCCCTGTATTCCGTCTCC	
T-bet	NM_019507.2	Forward: GCCTGGCGAGTTCTTCC	377
		Reverse: TGCTGCCTTCTGCCTTTC	
GATA3	NM_008091.3	Forward: GAAGGCATCCAGACCCGAAAC	255
		Reverse: ACCCATGGCGGTGACCATGC	
ROR-γt	NM_011281.2	Forward: AGGATGAGATTGCCCTCTACAC	121
		Reverse: AGATGATGATGGAAAGCCAGTT	

### Histopathological analysis

After the collection of BALF, the low right lobe of the lungs was collected and fixed in 4% (v/v) buffered formaldehyde and embedded in paraffin. Lung tissues were cut into 5 μm sections and then were stained with hematoxylin and eosin (H&E) or periodic acid Schiff (PAS), respectively. All images were captured using an Olympus DP50 digital camera (Olympus Optical Co.). Peribronchial inflammatory infiltration was assessed by a semiquantitative score (0–5) by 3 observers independently as described previously [28], and goblet cell hyperplasia with mucus hypersecretion was assessed with integrated optical density (IOD).

### Statistical analysis

Data were analyzed using the GraphPad software (version 5.0c). Data are presented as the mean ± standard deviation (SD). Differences between groups were analyzed using one-way analysis of variance (ANOVA) followed with the Tukey post hoc test. The significance level was defined as *p* < 0.05.

## Results

### Anti-IL-17A autoantibody induced by rMS-Ag85a-IL-17a *in vivo*

**Fig 2:**
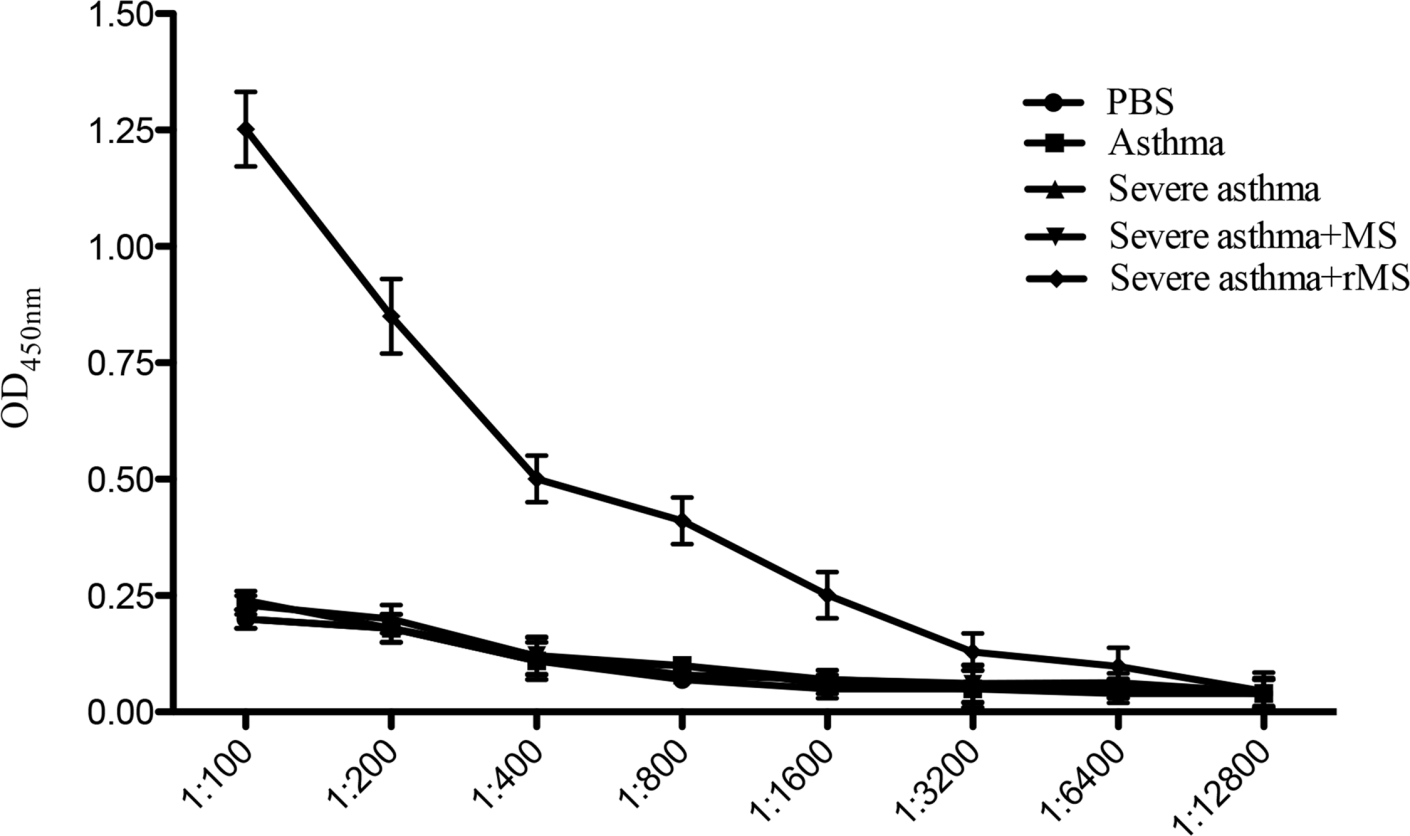
IL-17A autoantibody induced by rMS-Ag85a-IL-17a in sera. Sera were collected and serially diluted after the last challenge with OVA, anti-IL-17A specific IgG was measured by ELISA, and OD 450 nm showed the concentration of autoantibody specific to IL-17A in sera. Results are presented as mean value ± SD, n = 6.

In order to verify whether recombinant *Mycobacterium smegmatis* was able to induce anti-IL-17A autoantibody *in vivo*, an ELISA was performed and IL-17A protein was used as coating antigen to detect the specific IgG in sera. As (Fig 2) showed, sera from mice of PBS group, asthma group, severe asthma group and severe asthma plus MS had no IL-17A specific IgG, while mice treated with rMS-Ag85a-IL-17a had high titters of IgG specific to IL-17A in sera.

### Effect of rMS-Ag85a-IL-17a on allergic airway inflammation

**Fig 3:**
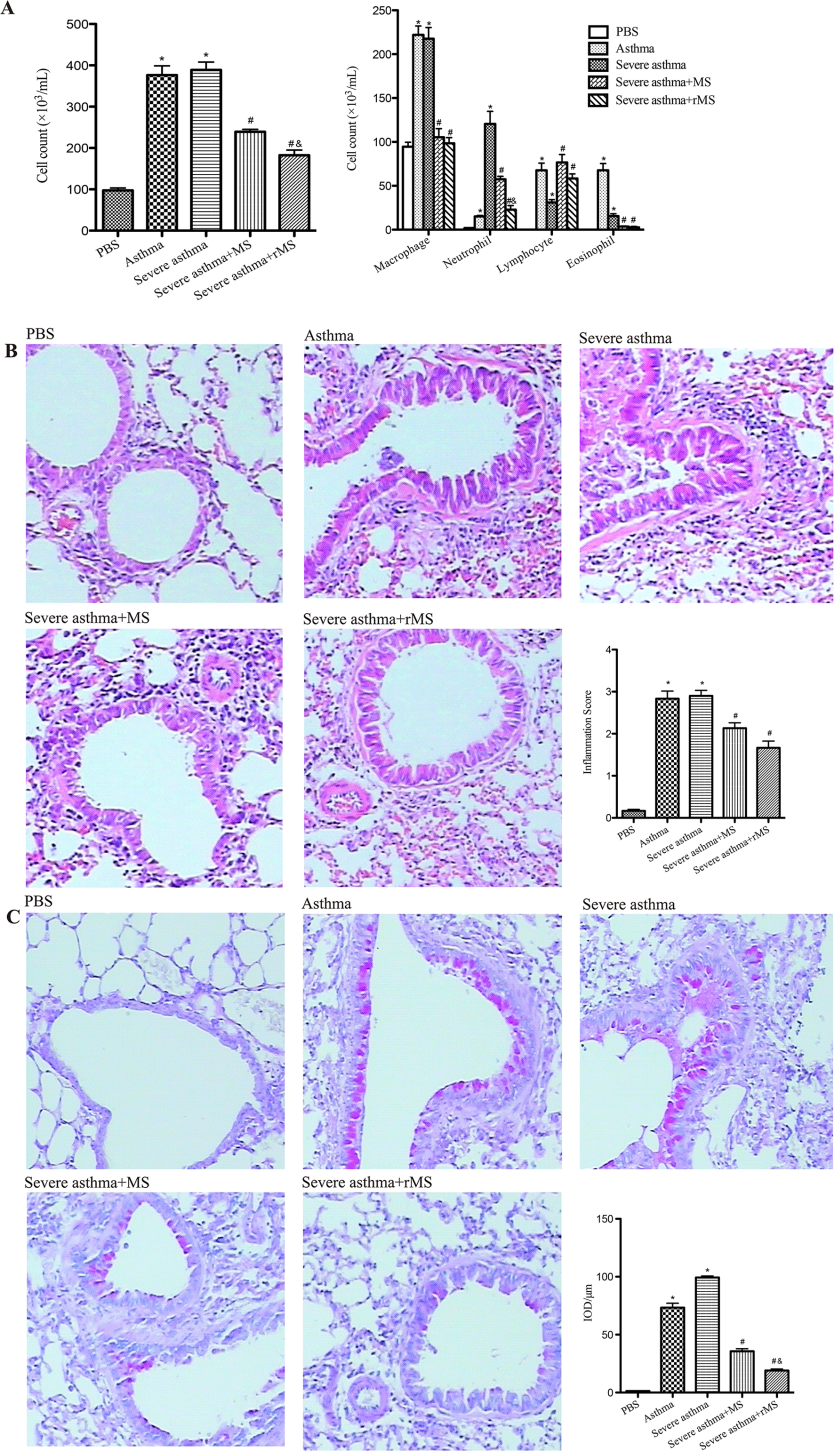
Effect of rMS-Ag85a-IL-17a on allergic airway inflammation. (A) The inflammatory cells in BALF were collected and stained with Wright-Giemsa, and then total cells and differential cellular components were count. (B) After the collection of BALF, lung tissue was fixed, sectioned at 2μm and stained with H&E or PAS (magnification×400). Lung inflammation was measured and defined as average of semiquantitative inflammation scores, and goblet cell hyperplasia was assessed with IOD. Results are presented as mean value ± SD, n = 6. *p < 0.01 vs. PBS group, #*p* < 0.01 vs. Severe asthma group, &*p* < 0.01 vs. Severe asthma plus MS group.

To investigate the effect of rMS-Ag85a-IL-17a on allergic airway inflammation, the inflammatory cells in BALF were collected and stained with Wright-Giemsa (Fig 3A). Compared with PBS group, the total leukocyte cells and macrophages in BALF were markedly elevated in asthma group and severe asthma group. Eosinophils in BALF of asthma group as well as neutrophils in BALF of severe asthma group were significantly increased compared with PBS group. These increases in the numbers of total leucocytes and neutrophils in BALF were significantly reduced by intranasal administration of rMS-Ag85a-IL-17a.

Further, histological analysis revealed that mice in model group (asthma and severe asthma) exhibited significantly increased peribronchial and perivascular inflammatory infiltration and marked goblet cell hyperplasia as compared with mice in PBS group (Fig 3B and Fig 3C). Moreover, the hyperplasia of goblet cell was markedly inhibited by rMS-Ag85a-IL-17a vaccination.

### Effect of rMS-Ag85a-IL-17a on the population of Th1 and Th2 cells in spleen

**Fig 4:**
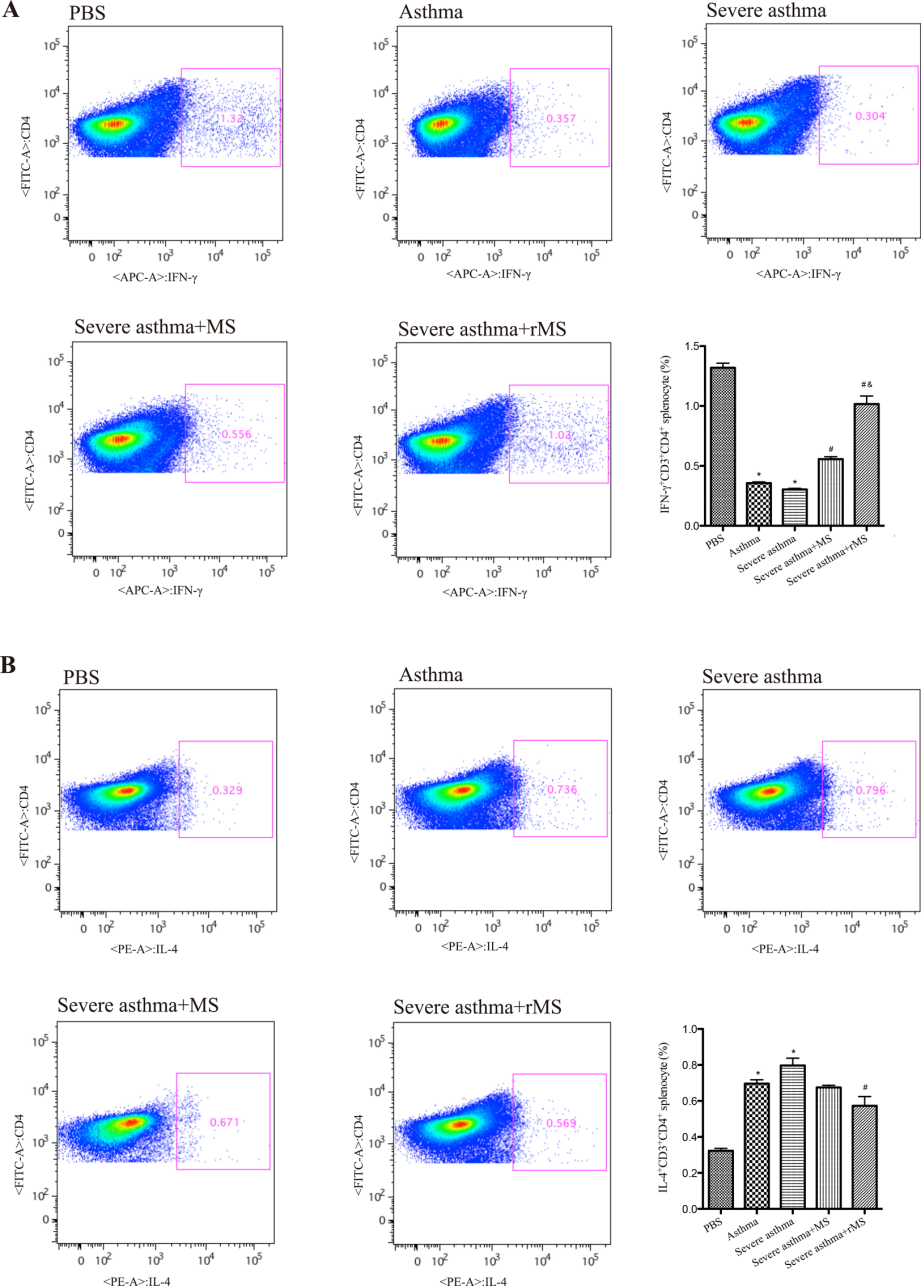
**Effect of rMS-Ag85a-IL-17a on IFN-γ+ (A) and IL-4+ (B) T cells in spleen.** Splenocytes were derived from mice and stimulated with OVA, after fixed and permeabilized, cells were incubated with extracellular and intracellular antibodies. Stained cells were run on a FACSCalibur cytometer and analyzed with FlowJo software. Results are presented as mean value ± SD, n = 6. *p < 0.01 vs. PBS group, #*p* < 0.01 vs. Severe asthma group, &*p* < 0.01 vs. Severe asthma plus MS group.

To determine whether recombinant *Mycobacterium smegmatis* affect the balance of Th1/Th2 cells, the population of IFN-γ^+^ Th1 cells and IL-4^+^ Th2 cells in spleen were evaluated. Following the last challenge, spleen cells were harvested and proportions of IFN-γ^+^ Th1 cells and IL-4^+^ Th2 cells were analyzed by flow cytometry. As shown in (Fig 4), the percentage of IFN-γ^+^ Th1 cells decreased from 1.32% in PBS group to 0.357% in asthma group and 0.304% in severe asthma group, while intranasal vaccination with recombinant *Mycobacterium smegmatis* significantly increased IFN-γ^+^ Th1 cells in spleen. The population of IL-4^+^ Th2 cells increased from 0.329% in PBS group to 0.736% in asthma group and 0.796% in severe asthma group, while recombinant *Mycobacterium smegmatis* vaccination significantly reduced IL-4^+^ Th2 cells in spleen.

### Cytokine levels in BALF and culture supernatant of splenocytes and mediastinal lymph nodes

**Fig 5:**
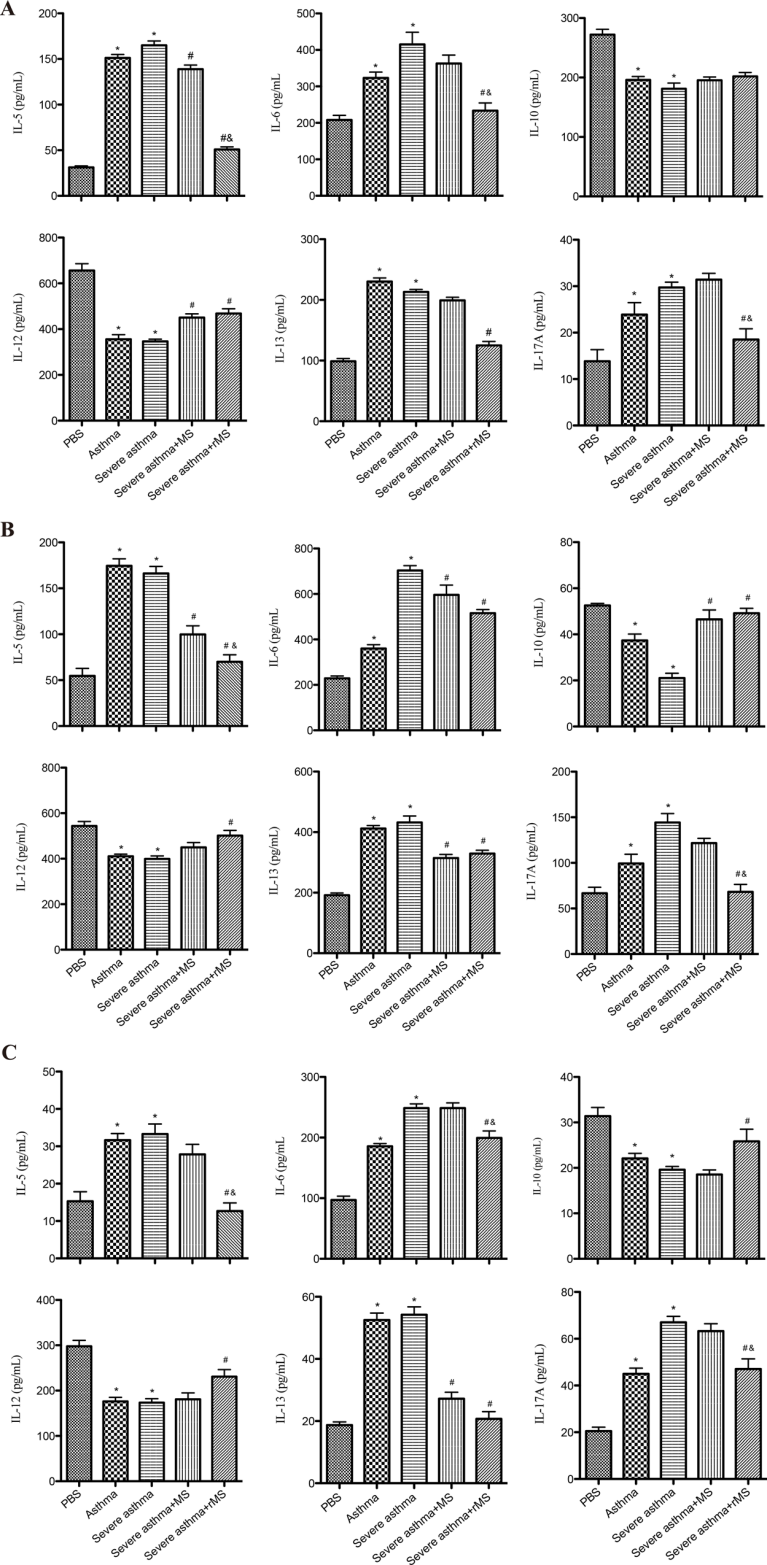
**Effect of rMS-Ag85a-IL-17a on cytokine levels in BALF (A) and culture supernatants of splenocytes (B) and mediastinal lymph nodes (C).** Results are presented as mean value ± SD, n = 6. *p < 0.01 vs. PBS group, #*p* < 0.01 vs. Severe asthma group, &*p* < 0.01 vs. Severe asthma plus MS group.

Furthermore, we assessed Th1, Th2 and Th17 cytokine levels in BALF and culture supernatant of splenocytes and mediastinal lymph nodes as shown in (Fig 5). ELISA showed that IL-5, IL-13, IL-6 and IL-17A levels in BALF and culture supernatant of splenocytes and mediastinal lymph nodes were significantly increased in model group, meanwhile levels of IL-10 and IL-12 in BALF and culture supernatant of splenocytes and mediastinal lymph nodes were significantly decreased in model group. These increases in levels of IL-5, IL-6 and IL-17A were significantly declined in rMS-Ag85a-IL-17a treated group.

### Effect of rMS-Ag85a-IL-17a on mRNA expression in lung tissue

**Fig 6:**
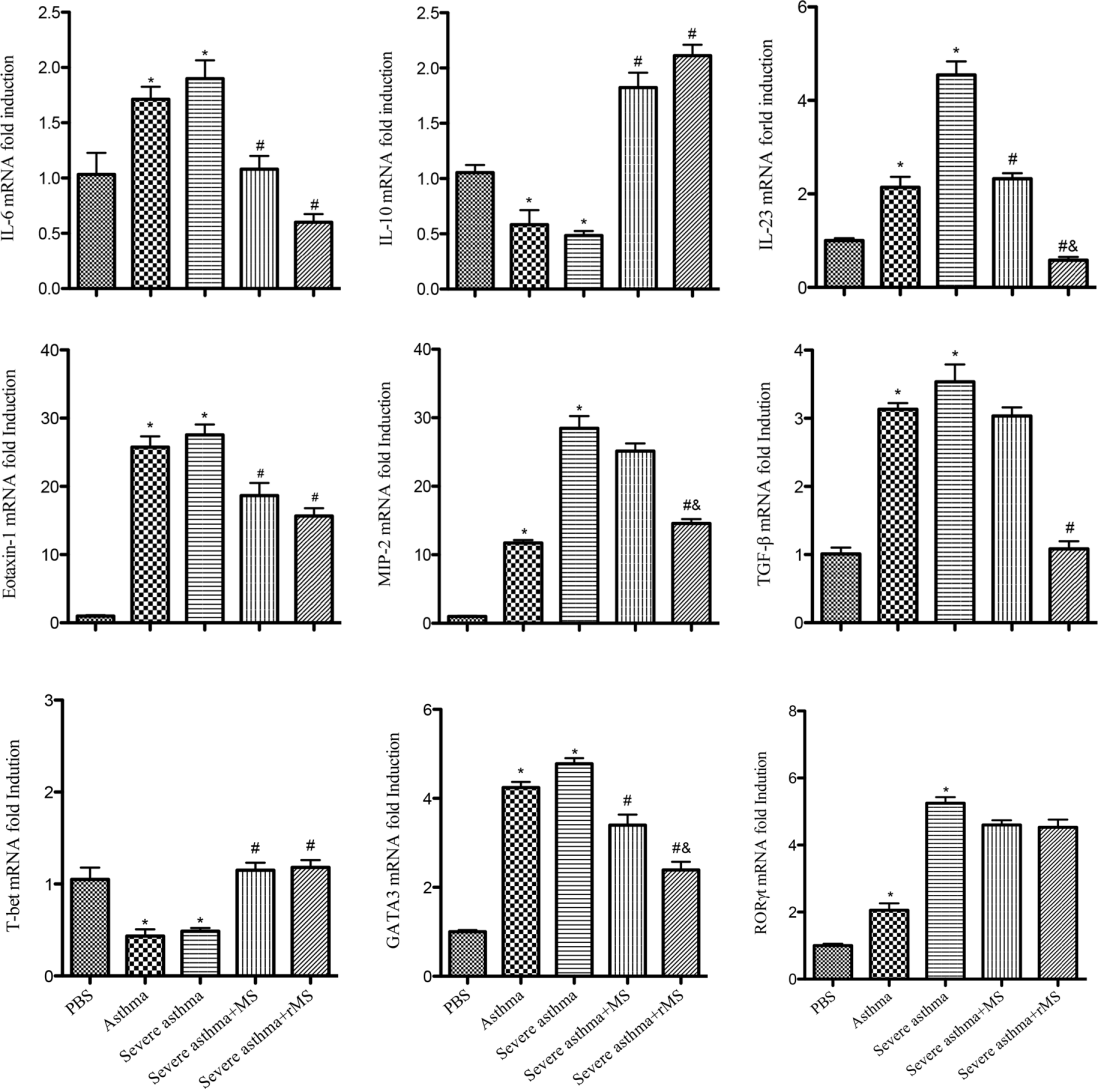
Effect of rMS-Ag85a-IL-17a on mRNA expression in lung tissue. Results are presented as mean value ± SD, n = 6. *p < 0.01 vs. PBS group, #*p* < 0.01 vs. Severe asthma group, &*p* < 0.01 vs. Severe asthma plus MS group.

We further assessed cytokine and chemokine mRNA levels of IL-6, IL-10, IL-23, Eotaxin-1, MIP-2, TGF-β as well as transcription factor mRNA levels of T-bet, GATA3 and RORγt in lung tissues. As shown in (Fig 6), compared with PBS group, mice in model group expressed significantly increased mRNA levels of IL-6, IL-23, Eotaxin-1, MIP-2, TGF-β, GATA3 and RORγt as well as decreased mRNA levels of IL-10 and T-bet. These increases of IL-23, MIP-2 and GATA3 were significantly declined in rMS-Ag85a-IL-17a vaccinated group; however, the administration of rMS-Ag85a-IL-17a did not affect RORγt expression in severe asthma.

## Discussion

Asthma is known as a chronic airway inflammatory disorder characterized by airway inflammatory cell infiltration, airway hyperresponsiveness, airway mucus secretion increased as well as airway remodeling, where inflammatory cells, airway structural cells and various cellular components are involved [29, 30]. Previous studies suggest that asthma is a Th2-type immune response dominated allergic disease, however, as Th17 cell subsets were found, Th1/Th2 immune imbalance theory is gradually being corrected. Accumulating evidence indicates that Th17 cell and Th17 cytokines such as IL-17A, IL-17F and IL-22 contribute to the development of asthma [31, 32]. As such, blocking or suppressing Th17 cell and their secretion of cytokines such as IL-17A appears to be a new direction in asthma therapy.

In order to overcome the disadvantages associated with using monoclonal antibodies or soluble receptors to block cytokines *in vivo*, strategies that use vaccines to induce autoantibodies neutralizing self-cytokines have begun to emerge [33, 34]. On the basis of our preliminary work, which confirmed that the recombinant *Mycobacterium smegmatis* induced anti-IL-17A autoantibody *in vivo*, in the present study, we further studied the protective functions of recombinant *Mycobacterium smegmatis* against asthmatic airway inflammation. Experimental results showed that nasal mucosa immunization with recombinant *Mycobacterium smegmatis* enhanced Th1-type immune response and decreased the number of Th2 cells and the expressions of IL-5, IL-13 and GATA3 as well as down-regulated the expressions of IL-6, IL-17A, Eotaxin-1 and MIP-2, furthermore inhibited the infiltration of inflammatory cells and airway mucus secretion, so that asthmatic airway inflammatory response was effectively attenuated.

Airway inflammation is the most important pathological hallmark of asthma, airway local accumulation and release of inflammatory cells and inflammatory mediators are the main causes of formation and maintenance of the airway inflammation leading to tissue damage and airway dysfunction, as such, effective suppression of airway inflammation is a key to alleviate asthma symptoms. It is well known that Th2 cytokines play a vital role in airway inflammation, especially in asthma with increased eosinophils and elevated serum IgE levels. Numerous studies have confirmed that Th2 cytokines participate in eosinophil differentiation, maturation and proliferation, and promote the secretion of IgE, meanwhile take part in the process of eosinophil cell aggregation in small airway by adjusting the functions of epithelial cells and smooth muscle cells, and inhibition of apoptosis of eosinophils [35–38]. In our study, the findings demonstrated that Th2 cell and cytokines, including IL-4, IL-5 and IL-13 were significantly increased in OVA-induced asthma model, while nasal mucosa immunization with recombinant *Mycobacterium smegmatis* significantly decreased the levels of IL-5 and IL-13 in BALF and splenocytes culture supernatant and mediastinal lymph nodes as well as down-regulated the mRNA expression of GATA3, furthermore the population of IL-4-producing Th2 cells was significantly decreased. Meanwhile, as Th1 cells are protective factor for allergic diseases, enhancing Th1-type immune response will undoubtedly be beneficial to reduce airway inflammation. It has been confirmed that IL-12 as a Th1 cytokines was a protective cytokine in asthma, which could promote the differentiation of Th1 cells and the production of IFN-γ, inhibit Th2 cells to secrete IL-4, IL-5 and IL-13, impact the differentiation of B cell, and inhibit the infiltration of eosinophil in airway [39]. In addition, clinical studies have confirmed IL-12 level was significantly decreased in asthma patients compared to normal objectives [40]. Our results showed that recombinant *Mycobacterium smegmatis* markedly elevated the number of IFN-γ-producing Th1 cells and the level of IL-12 in BALF and splenocytes culture supernatant and mediastinal lymph nodes as well as up-regulated the expression of T-bet in lung tissue, which could specifically promote Th0 cells into Th1 cells and inhibit the differentiation of Th2 cells [41]. From these, regulating Th1/Th2 immune balance is an important mechanism of recombinant *Mycobacterium smegmatis* to suppress airway inflammation.

With the in-depth study of asthma, it was found that the airway inflammation in asthmatic patients is not all dominated by eosinophil, for acute asthma, severe asthma and steroid-resistant asthma, airway inflammation manifested dominated neutrophil infiltration [42, 43]. In this experiment, OVA-induced asthmatic mice demonstrated obvious airway inflammation; the numbers of eosinophils, neutrophils and macrophages were significantly increased in BALF, histopathological analysis revealed that lung tissue from asthmatic mice had increased inflammatory cells infiltration and mucus secretion. On this basis, we established a severe asthma mouse model refereed to the experimental method of Hellings PW [15]. Our data revealed that airway inflammatory response in severe asthma group was significantly increased, which was characterized by dominated neutrophils accompanied by markedly elevated IL-17A and IL-6, and these inflammatory metrics were consistent with severe asthma and steroid-resistant asthma [44, 45].

Our results revealed that immunization with recombinant *Mycobacterium smegmatis* decreased the number of neutrophil in BALF and down-regulated the expressions of IL-17A and IL-6. Meanwhile, histopathological analysis revealed that recombinant *Mycobacterium smegmatis* significantly suppressed the infiltration of inflammatory cells and reduced airway mucus secretion. From these, the autoantibody of IL-17A induced by recombinant *Mycobacterium smegmatis* effectively inhibited the expression levels of IL-17A and IL-6, it not only could suppress the eosinophilic inflammation through regulating the Th1/Th2 balance, but also attenuate the neutrophilic airway inflammation in asthma.

Asthmatic airway inflammation is the main pathological feature, where a variety of inflammatory cells and mediators of inflammation involved, and chemokines play an important role in the formation and development of asthma through directed chemotaxis. Chemokines direct the migration of inflammatory cells to the bronchial mucosa and the recruitment of inflammatory cells via binding to receptors expressed on the cell surfaces of eosinophils, neutrophils, lymphocytes and macrophages. Moreover, chemokines could promote target cells to induce more chemokines, further exacerbating the inflammatory injury [46, 47]. Eotaxin-1, also known as CCL11, induces eosinophil precursor cells and mast cell maturation as well as promotes IL-4 and IL-5 secretion, MIP-2, also known as CXCL2, which involved in inflammation by directing and activating inflammatory cell, neutrophils are their specifically target cells [48–50], both of them play an important role in asthmatic airway inflammation. Our findings showed that recombinant *Mycobacterium smegmatis* effectively down-regulated the expressions of Eotaxin-1 and MIP-2 in lung tissues; meanwhile the numbers of eosinophils and neutrophils were significantly decreased in BALF. Thus, inhibition of chemokine expression could be another important mechanism of recombinant *Mycobacterium smegmatis* against airway inflammation in asthma.

In conclusion, recombinant *Mycobacterium smegmatis* induced anti-IL-17A autoantibodies *in vivo* and reduced IL-17A level, and further regulated Th1/Th2 immune balance and down-regulated the expression of chemokines, so that it effectively suppressed asthmatic airway inflammatory cells infiltration and mucus secretion, and attenuated airway inflammation. Hence, recombinant *Mycobacterium smegmatis* expressing Ag85A-IL-17A may be a new option for asthma treatment.

## Supporting Information

S1 Fig OD value of anti-IL-17A specific IgG in sera. (TIF)

S2 Fig The number of total leukocytes in BALF and the Differential cell counts. (TIF)

S3 Fig The semiquantitative scores of lung inflammation with HE staining. (TIF)

S4 Fig The IOD value of lung tissue with PAS staining. (TIF)

S5 Fig The percentages of IFN-γ+ (A) and IL-4+ (B) T cells in spleen.
(TIF)

S6 Fig Cytokine levels in BALF. (TIF)

S7 Fig Cytokine levels in culture supernatants of splenocytes. (TIF)

S8 Fig Cytokine levels in culture supernatants of mediastinal lymph nodes. (TIF)

S9 Fig Quantitative RT-PCR analysis of mRNA expression in lung tissue. (TIF)
