# Supplementary figures and images for: Intranasal Administration of Recombinant Mycobacterium smegmatis Inducing IL-17A Autoantibody Attenuates Airway Inflammation in a Murine Model of Allergic Asthma
Source: PLoS One. 2016 Mar 14;11(3):e0151581. doi: 10.1371/journal.pone.0151581 (PMC4790942; doi:10.1371/journal.pone.0151581)

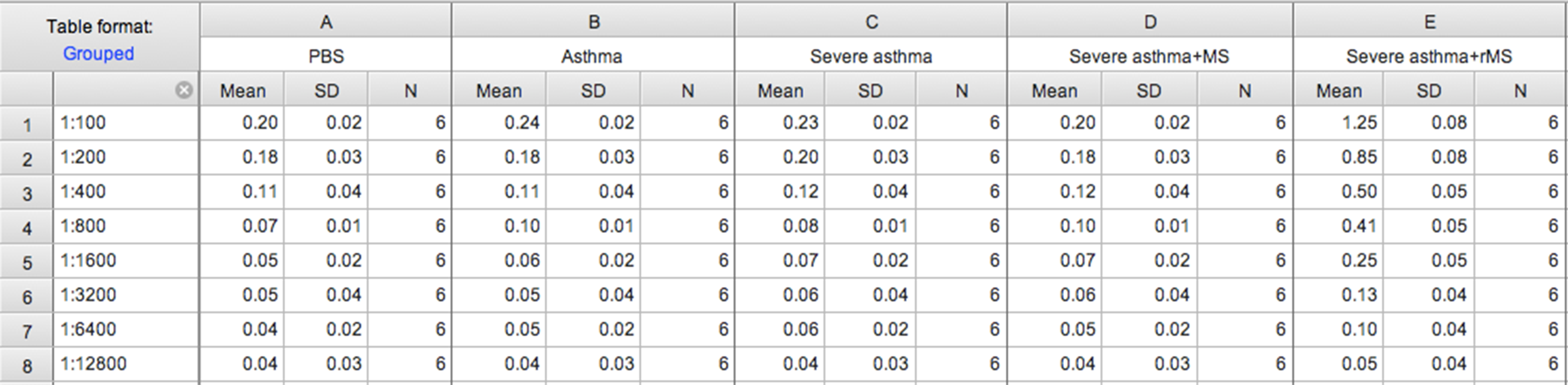

Supplement: S1 Fig — (TIF) [file pone.0151581.s001.tif]

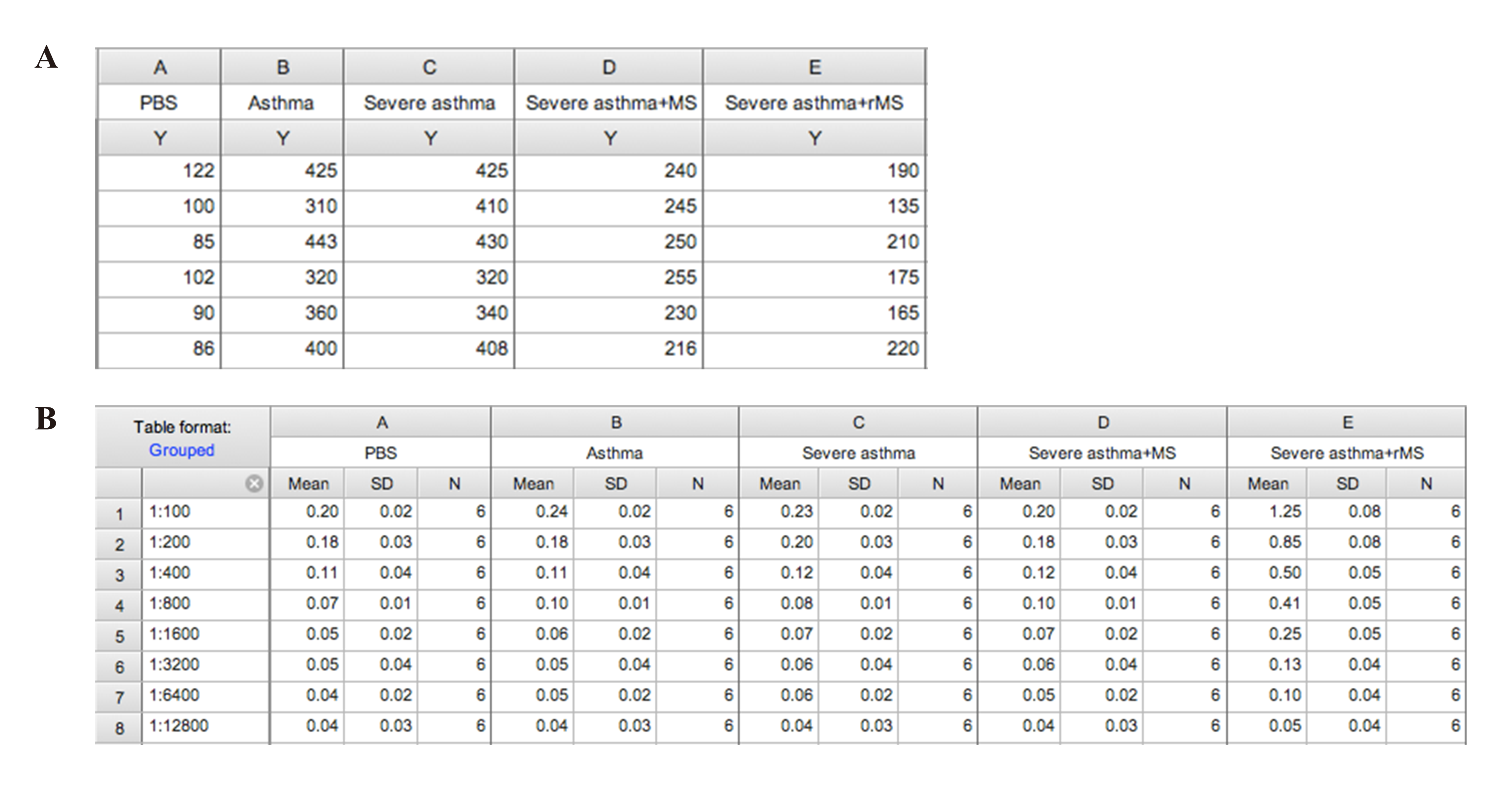

Supplement: S2 Fig — (TIF) [file pone.0151581.s002.tif]

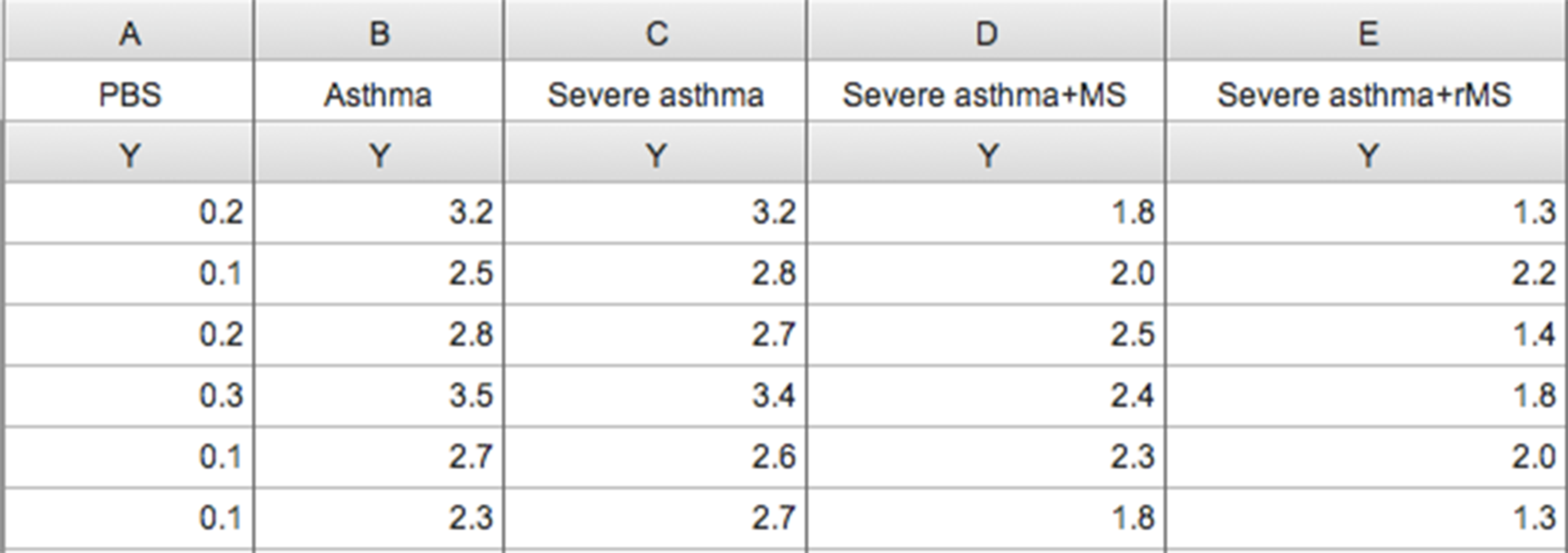

Supplement: S3 Fig — (TIF) [file pone.0151581.s003.tif]

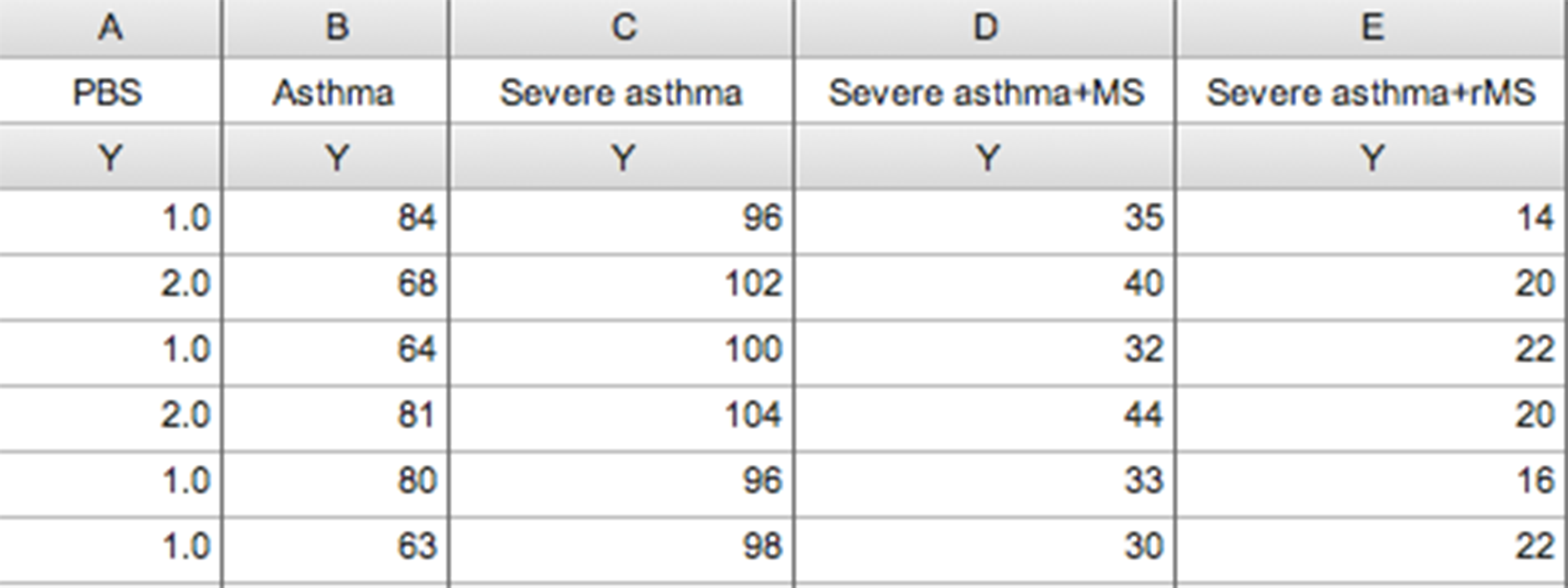

Supplement: S4 Fig — (TIF) [file pone.0151581.s004.tif]

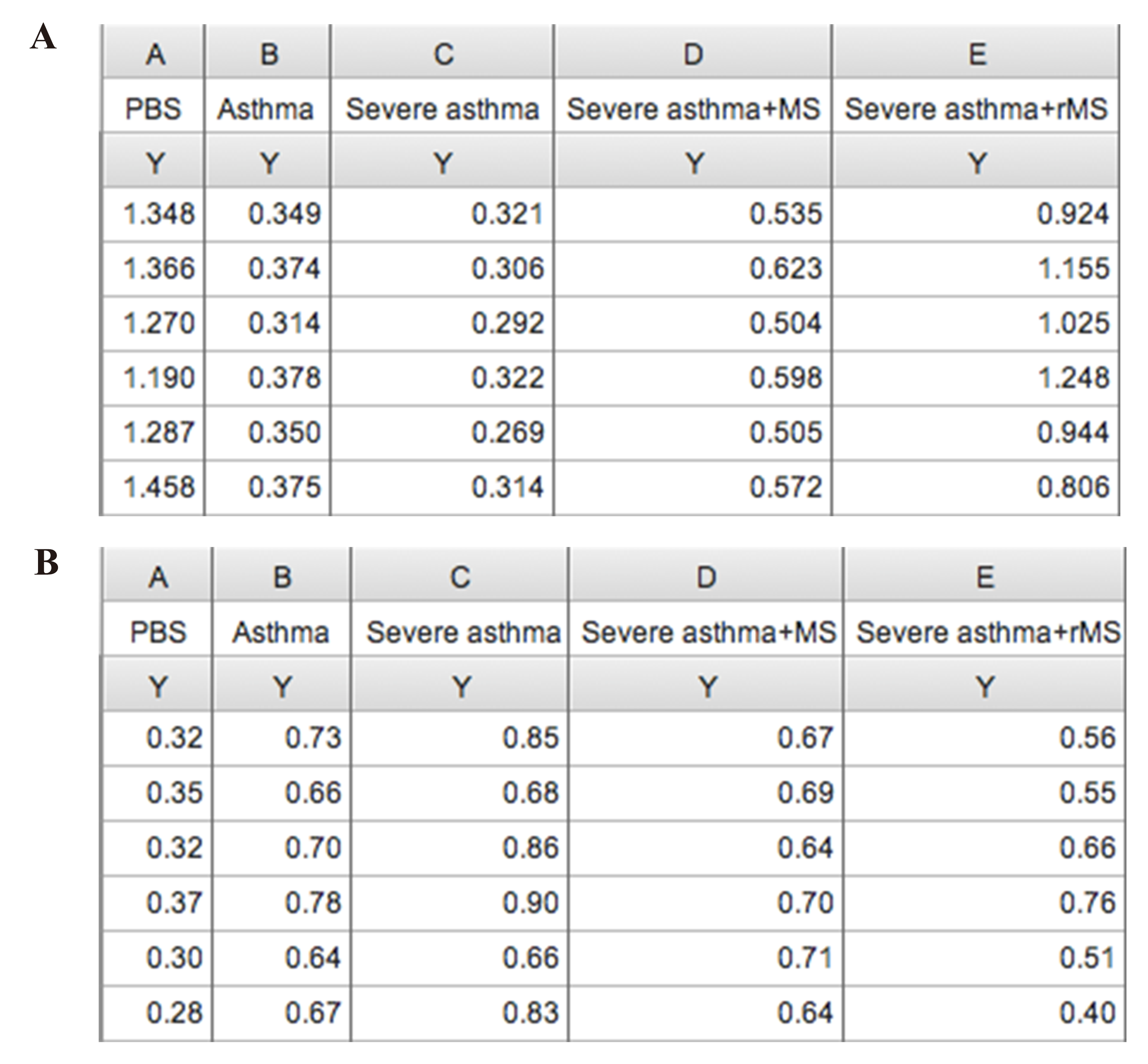

Supplement: S5 Fig — The percentages of IFN-γ+ (A) and IL-4+ (B) T cells in spleen. (TIF) [file pone.0151581.s005.tif]

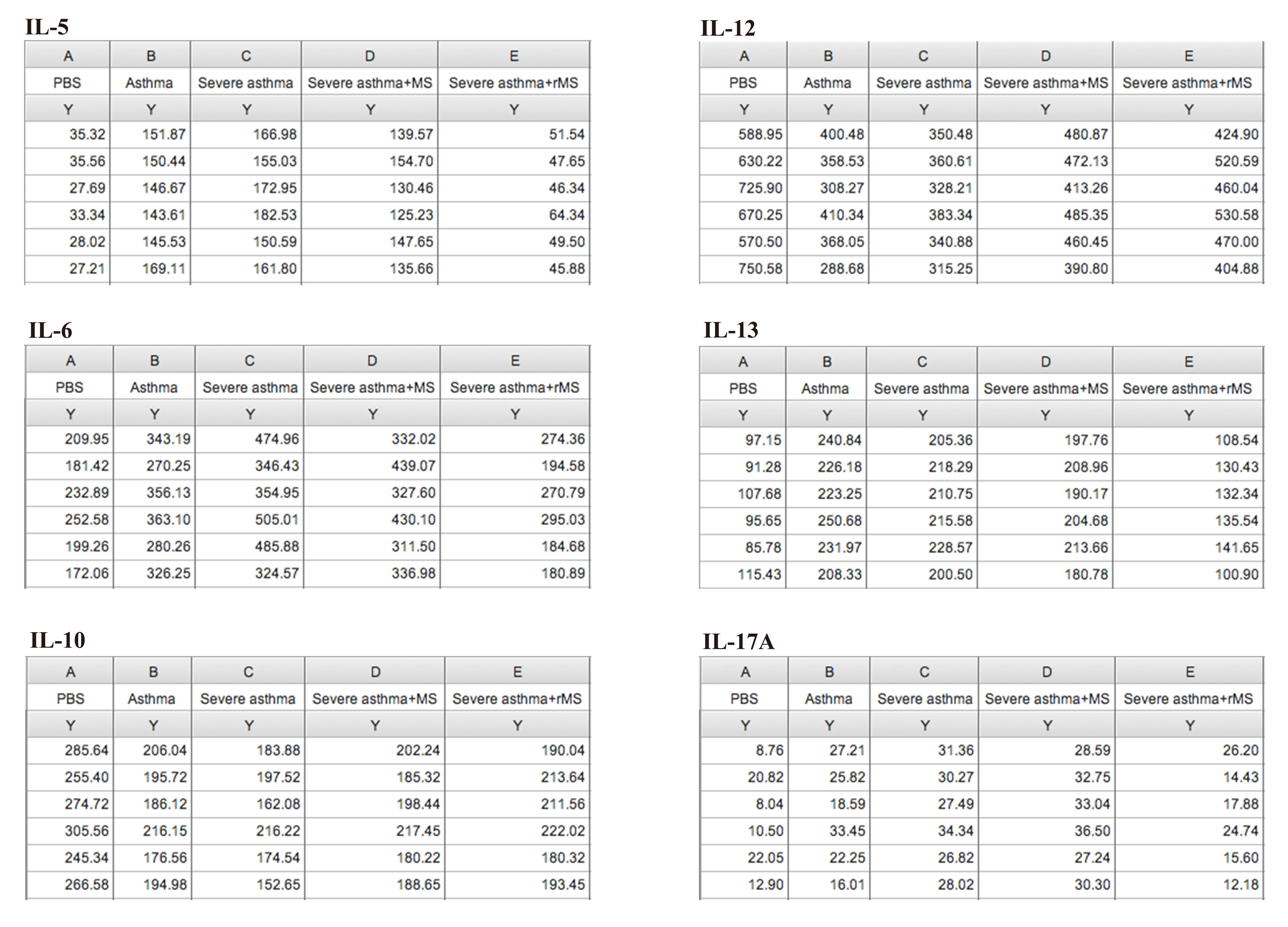

Supplement: S6 Fig — (TIF) [file pone.0151581.s006.tif]

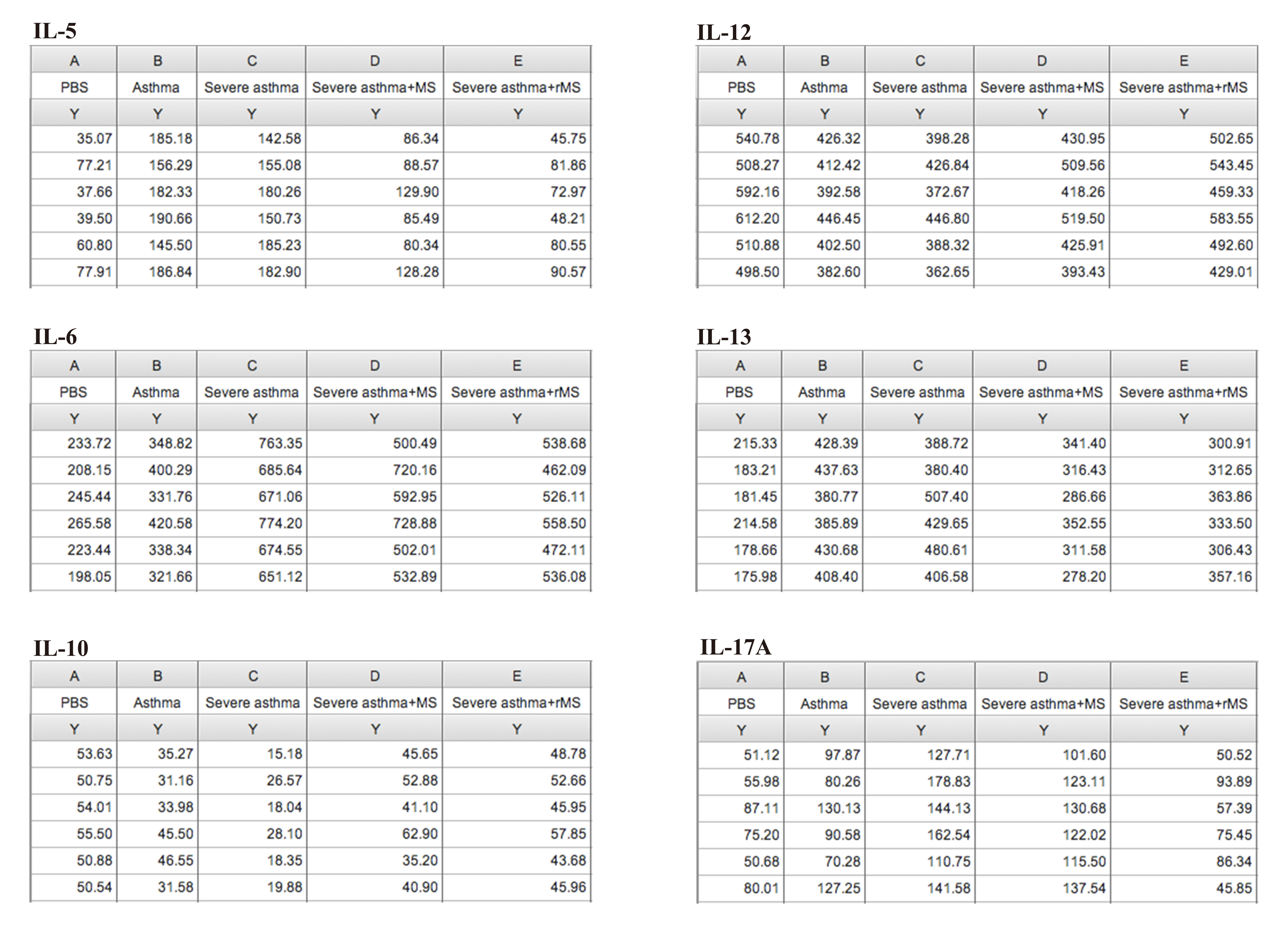

Supplement: S7 Fig — (TIF) [file pone.0151581.s007.tif]

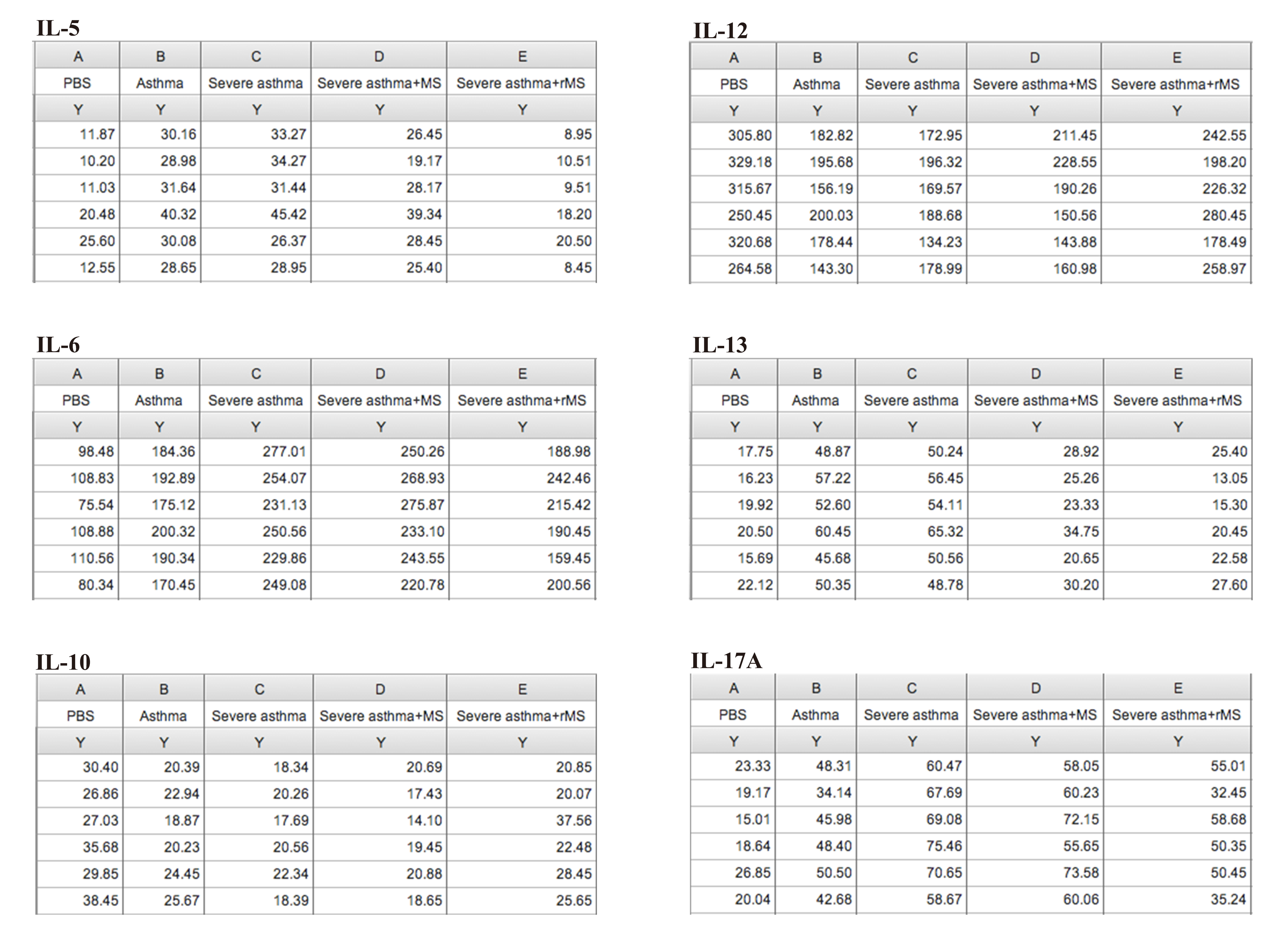

Supplement: S8 Fig — (TIF) [file pone.0151581.s008.tif]

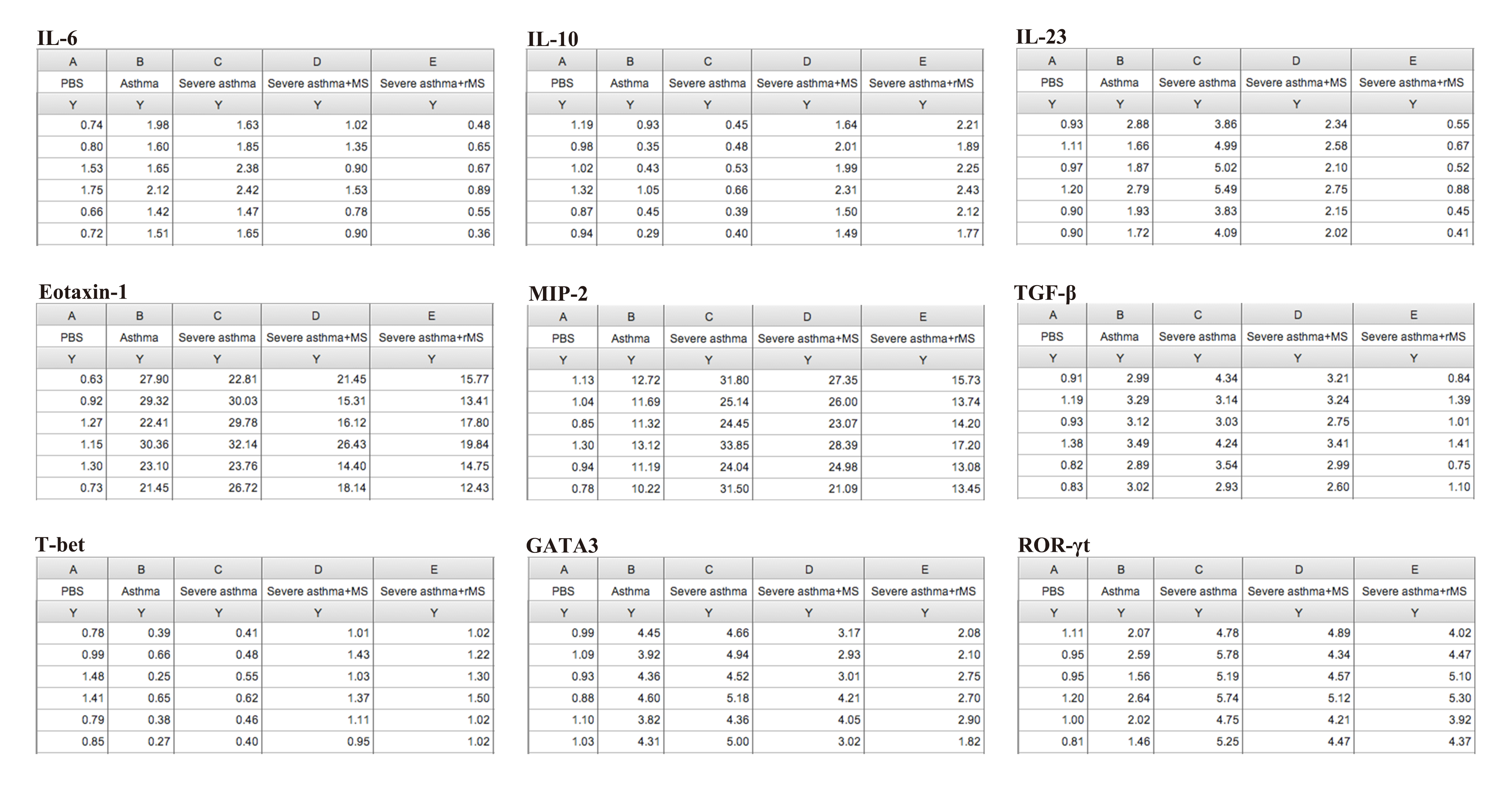

Supplement: S9 Fig — (TIF) [file pone.0151581.s009.tif]
